# ACCESS AND USAGE OF CARE AMONG OLDER MIGRANTS: CHALLENGES AND OPPORTUNITIES

**DOI:** 10.1093/geroni/igad104.1635

**Published:** 2023-12-21

**Authors:** Mushira Khan

**Affiliations:** Mather Institute, Evanston, Illinois, United States

## Abstract

Equitable access to care is critical to support healthy aging in underserved groups. Recent global demographic trends such as the rapid aging of the population in developing and developed countries, the growing proportion of migrants from the Global South to the Global North over the past 50 years, and increasing health inequalities between and within countries reflect a continued need to address the underlying pathways and causal mechanisms that determine access to and usage of care among migrants. This paper reviews the current state of knowledge on the micro-, meso-, and macro-level determinants that intersect and shape access to and usage of care services and programs among older migrants. It makes the case that foreign-born older adults are more likely to experience challenges accessing services and programs due to cultural and language barriers and factors related to their socio-economic status, race/ethnicity, gender, and time since immigration. Next, the paper provides a deeper exploration of the interface of structure and agency and an interrogation of how access and usage of care among older migrants may be conditioned, not simply by individual socio-economic circumstances and life course events, but also by macro-level factors like the distribution of power relationships and neoliberal public policies. The paper concludes with a call for sustained efforts to acquire a more profound understanding of individual-level, socio-economic and socio-political forces that influence access and usage of care. Finally, it provides directions for future research with the ultimate goal of supporting healthy aging among older migrants.

